# Workhouse Visiting and Management
**Recollections of Workhouse Visiting and Management*. By Louisa Twining. London: Kegan Paul, Trench and Co., Paternoster Square, E.C. Price 2*s*.


**Published:** 1887-02-05

**Authors:** 


### WORKHOUSE VISITING AND MANAGEMENT.*

There is hope for a nation which takes care of its sick and poor. When the perfunctory care of officials is stimulated and supplemented by the unsolicited attention and kindness of those who desire neither fee nor reward, the health of the body politic may be regarded as still more satisfactory. Such is approximately our condition to-day. In a little more than thirty years a complete revolution has been effected, and principally by the unwearying stedfastness of a few individuals. What would the world be if it were not for the few who constitute themselves human providences for the unthinking many.

Miss Twining, in a simple and unpretending volume tells the story of the workhouses: of those who dwelt and suffered in them; and of those who came to the rescue. Why should the pauper be helped? Was not he or she a paper because of his own incapacity or her own wickedness? Often, no doubt. But even so a wise humanity, and certainly an understood Christianity would not drive the fallen to deeper depths by contempt, but strive by pity and help to raise them up again. Besides, all paupers are not so by fault of their own. Many are *born* in workhouses; and still more in the ranks of those who are on the verge of pauperism. These children, through no fault of their own are found in the workhouse, and deserve no contempt, but unbounded pity. That which has happened to the pauper child might have happened to any of ourselves, even the greatest and the noblest. It is but an accident of birth. Alas, how sad an accident to those who must bear the consequences of it!

In her little book Miss Twining relates much of which this generation has no knowledge. She shows how the good and the bad were huddled together in one common mass in the workhouses. How the decent servant girl was compelled to associate with the abandoned women from the street. How the bringing of all these people together in one indiscriminate company produced a bold and hardened regiment of female paupers who were the terror of the officials, and the despair of those who sought their highest welfare. All these things are happily now past. The decent and the hopeful are separated from the abandoned, and have every encouragement to become respectable members of society. This great work has not been accomplished without a struggle. Officialism in its highest and lowest phases, from the President of the Poor Law Board to the most pompous Bumble has had to be met and managed. How it has all been done; and how Miss Twining and her many friends (both ladies and gentlemen) have set themselves with brave and generous hearts to help those who otherwise would have had no helper; is it not all written in the simple record of the experiences of twenty fruitful years? Miss Twining's book will not only be read with interest by those who know something of the questions at issue, but by all that large company who like to read of good work nobly undertaken, and of untold difficulties, one by one, met and overcome.

* *Recollections of Workhouse Visiting and Management.* By Louisa Twining. London: Kegan Paul, Trench and Co., Paternoster Square, E.C. Price 2*s*.

